# Lipo-Endomorphin-1 Derivatives with Systemic Activity against Neuropathic Pain without Producing Constipation

**DOI:** 10.1371/journal.pone.0041909

**Published:** 2012-08-17

**Authors:** Pegah Varamini, Friederike M. Mansfeld, Joanne T. Blanchfield, Bruce D. Wyse, Maree T. Smith, Istvan Toth

**Affiliations:** 1 School of Chemistry and Molecular Biosciences, The University of Queensland, Brisbane, Australia; 2 Centre for Integrated Preclinical Drug Development, The University of Queensland, Brisbane, Australia; 3 School of Pharmacy, The University of Queensland, Brisbane, Australia; Hokkaido University, Japan

## Abstract

To enhance the drug-like properties of the endogenous opioid peptide endomorphin-1 (**1** = Tyr-Pro-Trp-Phe-NH_2_), the N-terminus of the peptide was modified with 2-aminodecanoic acid, resulting in compound **3**. Tyr in compound **1** was replaced with 2,6-dimethyltyrosine yielding compound **2**. Derivative **2** was also substituted with 2-aminodecanoic acid producing compound, **4**. Lipoamino acid-modified derivatives showed improved metabolic stability and membrane permeability while maintaining high μ-opioid (MOP) receptor binding affinity and acting as a potent agonist. *In vivo* studies showed dose-dependent antinociceptive activity following intravenous (i.v.) administration of compounds **3** and **4** in a chronic constriction injury (CCI)-rat model of neuropathic pain with ED_50_ values of 1.22 (±0.93) and 0.99 (±0.89) µmol/kg, respectively. Pre-treatment of animals with naloxone hydrochloride significantly attenuated the anti-neuropathic effects of compound **3**, confirming the key role of opioid receptors in mediating antinociception. In contrast to morphine, no significant constipation was produced following i.v. administration of compound **3** at 16 µmol/kg. Furthermore, following chronic administration of equi-potent doses of compound **3** and morphine to rats, there was less antinociceptive tolerance for compound **3** compared with morphine.

## Introduction

Centrally acting opioids, such as morphine, are the most frequently used analgesic agents for the treatment of severe nociceptive pain but they also produce a range of unpleasant adverse effects including constipation, analgesic tolerance and cardio-respiratory depression [1]. In addition, morphine exhibits poor efficacy against neuropathic pain. Neuropathic pain is a condition associated with nerve damage or diseases affecting the somatosensory system [2]. Current medications do not confer enough efficacy to alleviate neuropathic pain, producing pain relief in less than 50% of patients [3]. Two endogenous opioid peptides, endomorphin-1 (Tyr-Pro-Trp-Phe-NH_2_) and endomorphin-2 (Tyr-Pro-Phe-Phe-NH_2_) have been extensively investigated to be used as potential analgesics in place of morphine [4]. They exhibit high selectivity, affinity and agonist activity at μ-opioid (MOP) receptors [5], receptors through which their selective agonists display the highest antinociception [6]. Endomorphins have been shown to produce potent antinociception in rodent models of acute [7] and neuropathic pain after central administration [8] with less undesirable side effects than opioid alkaloids. Widespread laboratory investigations carried out on the possible use of endomorphins in place of morphine-like analgesics, however, were unsuccessful. Like all small peptides, endomorphins suffer from a short duration of action even after central administration (5–15 min) [9], [10], poor metabolic stability, and the inability to cross the gut mucosa and the blood-brain barrier (BBB). To be of use for clinical application, it is essential to improve their membrane permeability, resistance to enzymatic degradation, and ability to enter the central nervous system (CNS). Various chemical modifications to the structure of the endomorphins have been investigated to enhance the bioavailability. These include unnatural amino acid substitution [11], β-amino acid substitution [12], cyclization [13], and synthesis of bivalent ligands [14].

Previously, we showed that incorporation of lipoamino acids (LAAs), which combine the structural features of lipids and amino acids, into the structure of short peptides can improve the metabolic stability and membrane permeability of endomorphins [15]. LAAs are lipophilic due to their alkyl side chains but have a polar head group and exhibit the conformational characteristics of amino acids. This makes them ideal candidates for increasing the lipophilicity of small peptides with a subsequent increase in their passive diffusion across plasma membranes without altering their structure or functionality [16]. In this study, we applied this approach to stabilize the structure of endomorphin-1 and reduce its vulnerability to proteolysis whilst retaining the anti-neuropathic potency after systemic administration. As C-terminal modification of endomorphins has previously been shown to cause considerable reduction in receptor binding affinity [15], we modified the N-terminus of endomorphin-1 using a 10-carbon lipoamino acid residue, 2-aminodecanoic acid. Variations in alkyl chain length of the LAA can be employed for tuning the physico-chemical properties of the resulting peptide analog [17]. We employed racemic d,l-2-aminodecanoic acid to obtain stability while maintaining solubility of the diastereomeric products. Tyr^1^ was replaced in two of the endomorphin-1 derivatives with an unnatural amino acid, 2,6-dimethyltyrosine (Dmt). Dmt was previously shown to enhance μ-opioid (MOP) receptor binding affinity and potency of several opioid peptides [18] including endomorphin-1 [11]. The resulting peptides were studied for their *in vitro* biological activity including stability, permeability, and MOP receptor binding activity, as well as *in vivo* antinociceptive activity in a chronic constriction injury (CCI) model of neuropathic pain in rats. The LAA-modified analog bearing the natural amino acid Tyr was further characterized for unwanted pharmacological activities including its propensity to induce constipation and induce antinociceptive tolerance in rats. We also confirmed the involvement of opioid receptors using the non-selective opioid receptor antagonist, naloxone hydrochloride.

## Results

### Chemistry

**Figure 1:**
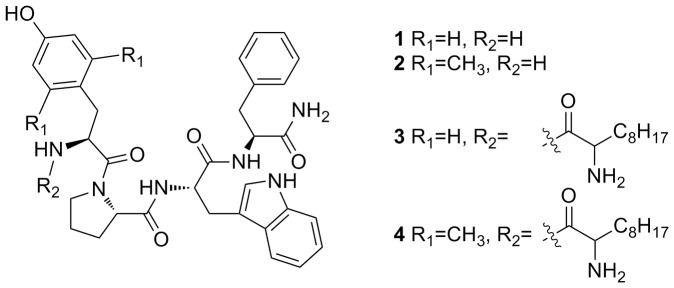
Structure of endomorphin-1 (1) and its derivatives (2–4).

All endomorphin analogs, **1–4** (Fig. 1), were synthesized by solid phase peptide synthesis (Scheme S1) and purified to a single peak (>95% purity) by analytical RP-HPLC. Peptides were characterized by ESI-MS. Purified yields of the compounds were 84% (108 mg) for the native endomorphin-1 (**1**), 57% (86 mg) for compound **2**, 43% (466 mg) for compound **3** and 40% (450 mg) for compound **4**.

### Metabolic stability and membrane permeability

*In vitro* stability and permeability of the peptide analogs were evaluated using the Caco-2 cell line derived from human colorectal carcinoma cells. Caco-2 cells are widely used as an *in vitro* model system to evaluate enzymatic stability of a compound after systemic or oral administration and also as a preliminary gut-blood and BBB permeability screening tool [19]. Furthermore, plasma stability of the compounds was evaluated in human plasma.

Compound **1** degraded in the Caco-2 cell homogenate and plasma, respectively (Table 1). Dmt-modified peptide **2** showed a moderate increase in Caco-2 cell homogenate stability with t_1/2_ of 24.1 (±3.6) min but only a slight increase in plasma stability (t_1/2_ = 4.2 (±1.5) min). In contrast, N-terminal modification of the peptide with LAA in compounds **3** and **4** significantly increased the Caco-2 cell homogenate stability 15- and 34-fold compared with the corresponding parent peptide **1** tested in the same experiment (*p*<0.05). The half-lives for compounds **3** and **4** were obtained at 77.4 (±7.1) and 96.3 (±8.3) min, respectively, Table 1). The plasma stability observed for compound **4** showed a 19-fold increase in half-life (t_1/2_ = 41.4 (±3.3) min), relative to peptide **1** (Table 1).

**Table 1:** Summary of *in vitro* results.

Compound	*t*_1/2_ (min, Caco-2)	*t*_1/2_ (min, Plasma)	*P*_app_ (×10^−7^, cm/s)[a]	*K*_iμ_ (nM)[b]	cAMP, IC_50_ (nM)[c]
**1**	5.0±1.2	2.70±0.9	0.2±0.02	1.1±0.2	14.0±6.8
	2.3±0.36[d]		1.0±0.34[d]	(1.1±0.09)[d]	(9.4±0.43)[d]
**2**	24.1±3.6	4.2±1.5	2.5±0.3	0.06±0.01	0.3±0.02
**3**	77.4±7.1[d]*	ND	90.1±12.2[d]*	25.7±1.3[d]	31.4±1.45[d]
**4**	96.3±8.3*	41.4±3.3*	12.4±4.7*	0.47±0.12	1.30±0.22

[a] The apparent permeability, *P*_app_ (cm/s), across Caco-2 cell monolayers. Analysis were done using LC/MS and each point is expressed as mean ± SEM.

**Figure 2:**
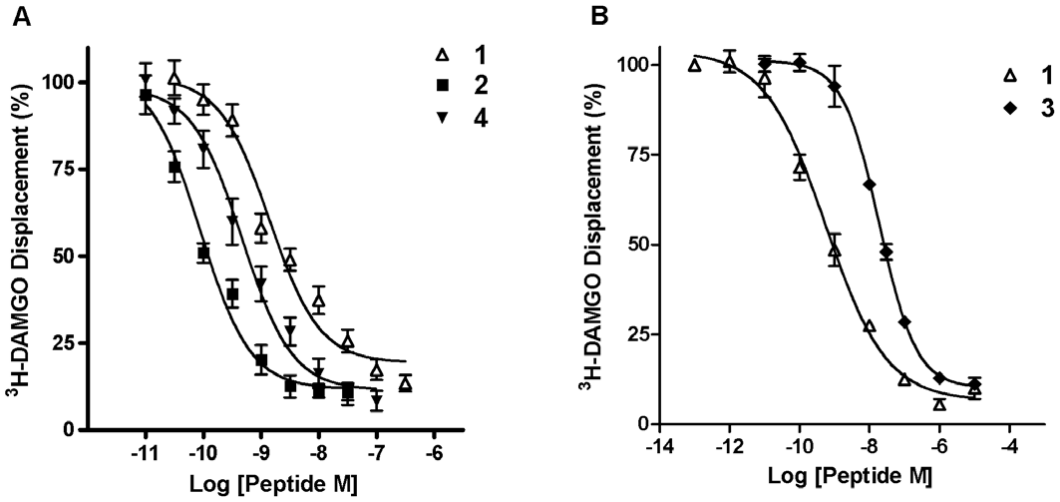
Competitive radioligand binding of endomorphin-1 and its analogs to MOP receptors. Binding affinity of compound A) **1**, **2**, and **4**; B) **1,** and **3** determined by competitive displacement of [^3^H]DAMGO from whole SH-SY5Y cells. Data presented are mean (± SEM) from three separate experiments, each performed in triplicate.

[b] The *K*_iμ_ values of **1**, **2**, **3** and **4** for the MOP receptor were obtained using competitive radioligand binding assay. Competitive displacement of the MOP selective radioligand, [^3^H]DAMGO, was determined from whole SH-SY5Y cells in the presence of DPDPE (1 µM). Binding affinity values (*K*_iμ_) were determined using seven concentrations of each compound in the range 10 pM to 1 µM performed in three independent experiments, each in triplicate. Data expressed as mean ± SEM. Competitive binding curves are shown in Fig. 2.

**Figure 3:**
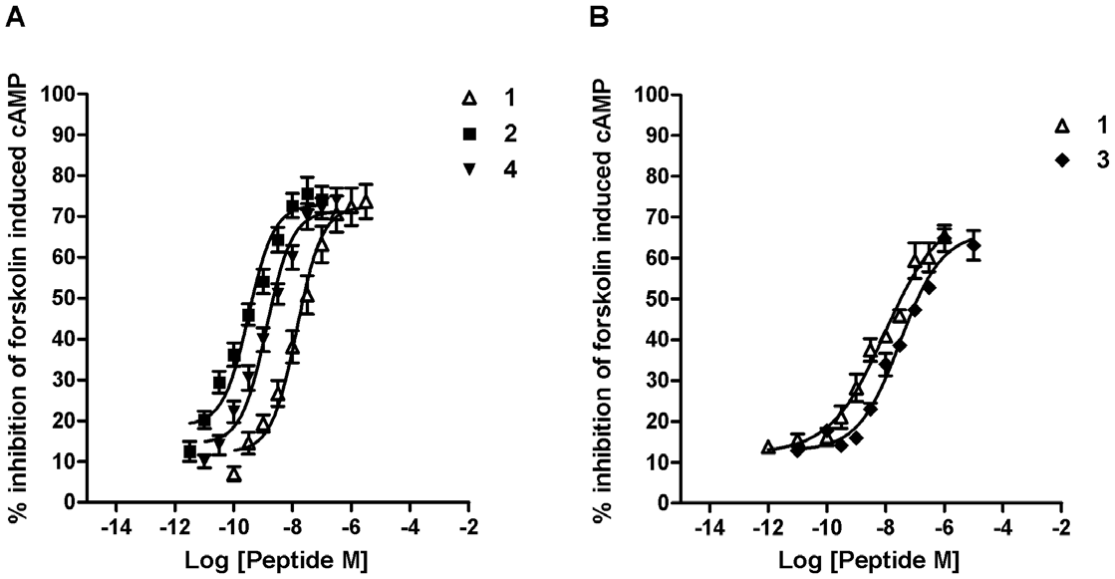
Inhibition of forskolin-induced cAMP formation. Dose-response curves for the inhibitory effects of compounds A) **1**, **2**, and **4**; B) **1**, and **3** on forskolin-stimulated cAMP formation in SH-SY5Y cells. Data presented are mean (± SEM) from three separate experiments, each performed in triplicate.

[c] The IC_50_ values (nM) were estimated from dose-response curves shown in Fig. 3. Inhibition of forskolin-stimulated cAMP formation was measured to determine the agonist activity of the compounds. Experiments were performed three independent times, each in triplicate.

[d] These values are obtained from the same experiment. ND, not determined. Each value represents mean ± SEM. *p<0.05, compared to compound **1** in the same experiment.

*In vitro* permeability of the endomorphin-1 analogs was investigated using Caco-2 cell monolayers. Endomorphin-1 (**1**) and compound **2** showed low permeability across Caco-2 cell monolayers with an apparent permeability (*P*_app_) of 0.2 (±0.02)×10^−7^ cm/s and 2.5 (±0.3)×10^−7^ cm/s. In contrast, the LAA-modified analog **4** displayed a 62-fold increase in permeability with *P*_app_ 12.4 (±4.7)×10^−7^ cm/s (Table 1, *p*<0.05). Analog **3** was examined in a separate experiment. Native peptide **1** was tested along with compound **3** to allow direct comparison. Compound **3** displayed a significant permeability through Caco-2 cell monolayers with *P*_app_ 90.1 (±12.2)×10^−7^ cm/s, an ∼90-fold increase compared to the *P*_app_ of peptide **1**, tested in the same experiment (Table 1, *p*<0.05).

### Receptor binding and functional activity

Opioid binding affinity and *in vitro* agonist activity of endomorphin-1 analogs were investigated using a human neuroblastoma cell line, SH-SY5Y cells, which endogenously expresses MOP and δ-opioid (DOP) receptors. To test the agonist behavior of the peptide derivatives, accumulation of cyclic adenosine monophosphate (cAMP) was evaluated upon stimulation with forskolin in SH-SY5Y cells. Forskolin-stimulated formation of intracellular cAMP is reduced by MOP receptor agonists [20].

Unlike compound **3**, which exhibited a 23-fold decrease in opioid receptor affinity, compound **4** showed slightly higher MOP receptor binding affinity (*K*_iμ_ 0.5 (±0.1) nM) compared with the native peptide, **1**, (*K*_iμ_ 1.1 (±0.2) nM, Table 1 and Fig. 2A, 2B). Peptides **3** and **4** showed MOP receptor agonist activity by inhibiting forskolin-stimulated cAMP formation in a concentration-dependent manner. Although IC_50_ values for compound **3** increased slightly, replacement of Tyr with Dmt in compound **4** enhanced agonist potency compared to compound **1,** exhibiting a decrease in IC_50_ from 14.0 (±6.8) nM for endomorphin-1 to 1.3 (±0.2) nM for compound **4** (Table 1 and Fig. 3A, 3B).

### Antinociceptive activity

**Figure 4:**
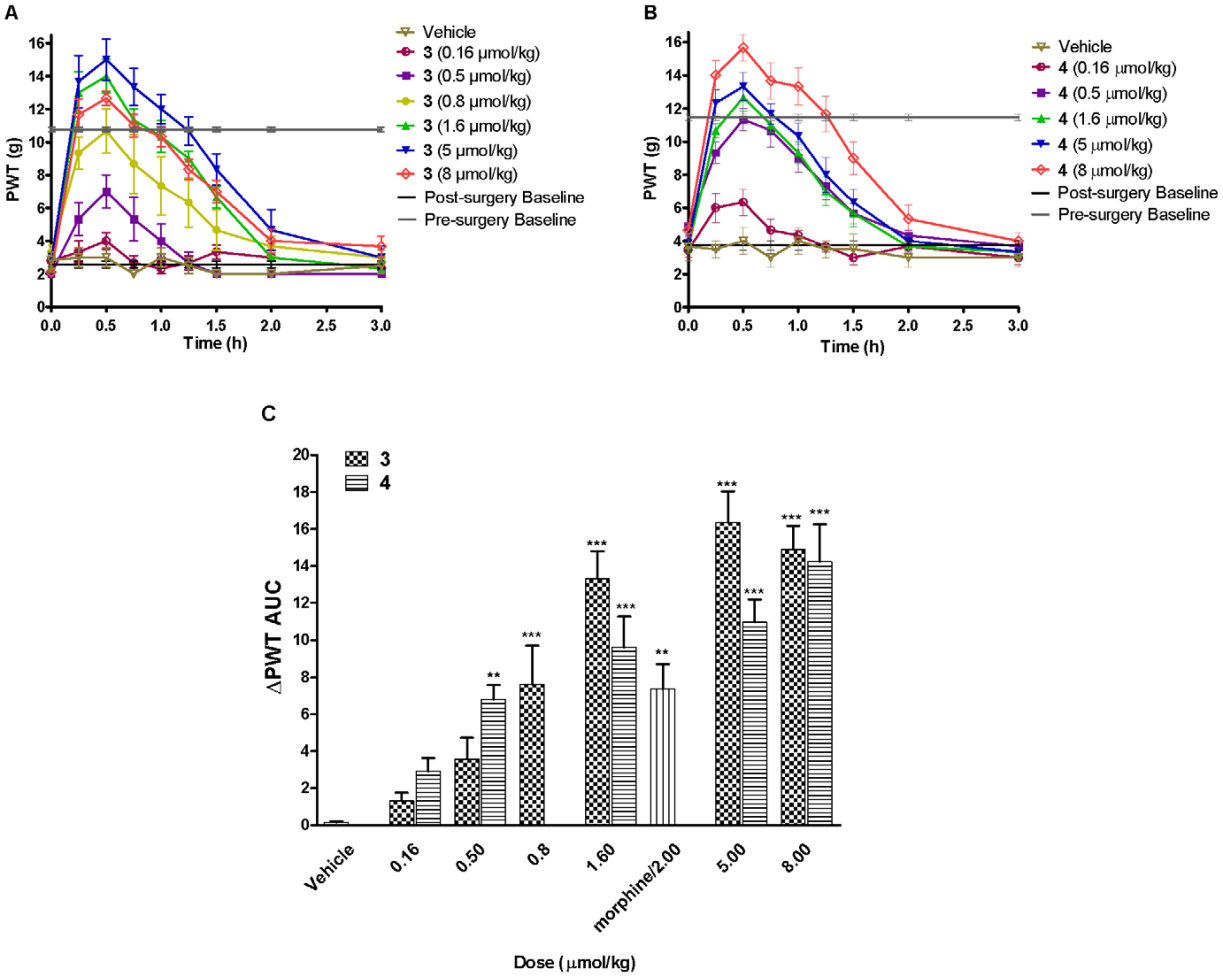
Intravenous dosing of compounds 3 and 4 in the ipsilateral hindpaw of CCI-rats. Paw withdrawal threshold (PWT) versus time curves for single bolus i.v. doses of compounds A) **3;** B) **4**, and vehicle in CCI-rats; C) The extent and duration of antinociception (areas under the ΔPWT versus time curves, ΔPWT AUC values) produced by single i.v. bolus doses of compounds **3** and **4** at 0.8 and 0.5 µmol/kg, respectively, was significantly greater than that produced by vehicle. This effect was comparable with the effect of morphine at 2 µmol/kg. Each value represents mean (± SEM). **p<0.01, ***p<0.001 compounds **3** and **4** *vs*. vehicle treated CCI-rats (*n* = 6–8).

**Figure 5:**
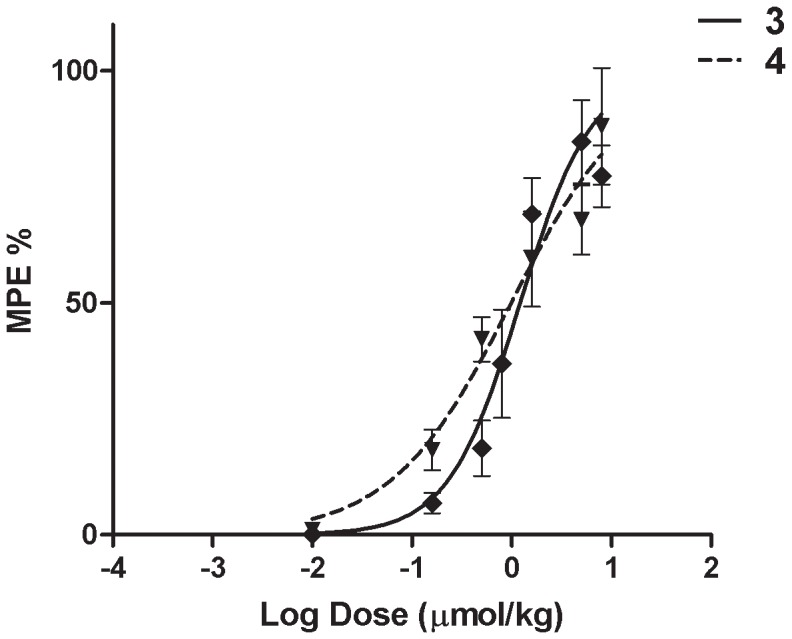
Antinociceptive dose-response curves for compounds 3 and 4. Analogs **3** and **4** produced dose-dependent analgesia after single i.v. bolus administration to CCI-rats. The data are mean (± SEM).

**Figure 6:**
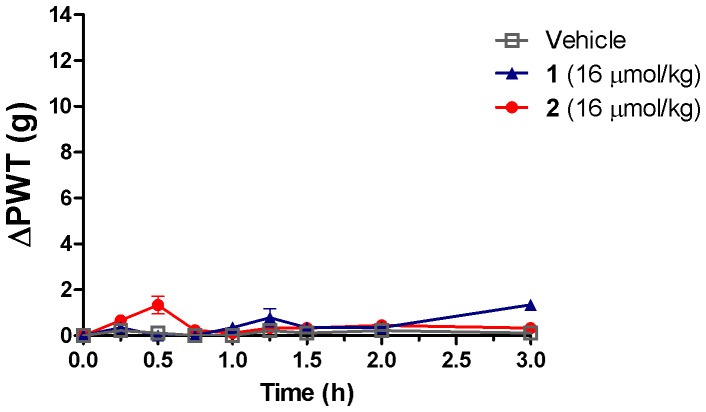
Insignificant antinociception produced by compounds 1 and 2. The highest doses of compounds **1** and **2** did not produce pain relief at 16 µmol/kg after single bolus i.v. administration to CCI-rats. Each value represents mean (± SEM) (*n* = 6–8).

**Figure 7:**
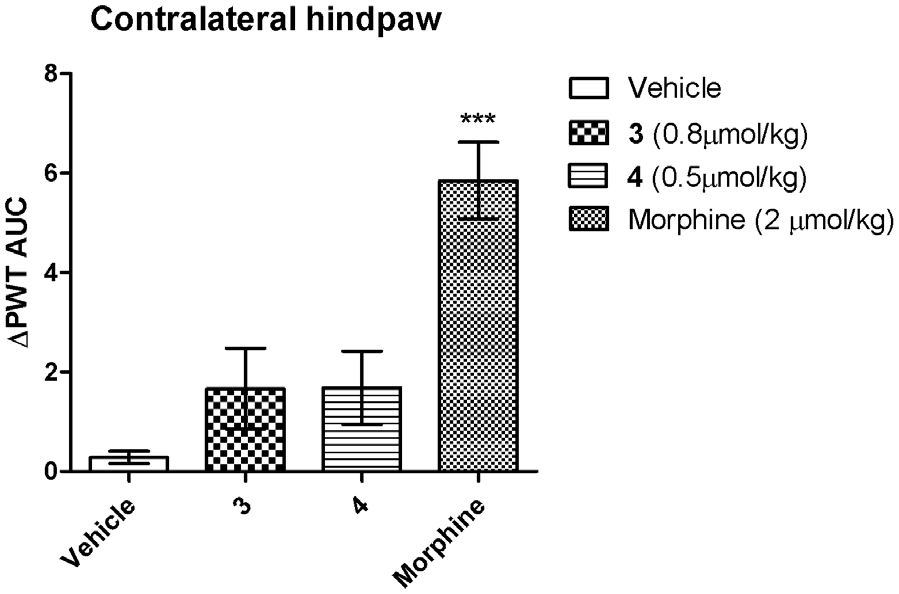
Extent and duration of antinociception in the contralateral hindpaw of CCI-rats. Single i.v. bolus doses of compounds **3** and **4** produced insignificant anti-allodynic responses in the contralateral hindpaws of CCI-rats in contrast to morphine at equi-effective doses. Each value is the mean (± SEM). ***p<0.001, *vs.* vehicle treated CCI-rats, *p*>0.05 for compounds **3** and **4** *vs.* vehicle (*n* = 6–8).

To investigate if the stabilization of endomorphin-1 by LAA conjugation resulted in active analogs following systemic administration, the comparative antinociceptive effects of parent peptide **1** and derivatives **2–4** were assessed using the CCI-rat model of neuropathic pain [21]. Endomorphin-1, **1**, and derivatives **2–4** were administered intravenously (i.v.) as single bolus doses, from 0.16 µmol/kg to 16 µmol/kg. Compounds **3** and **4** produced dose-dependent anti-allodynic activity in the ipsilateral (injured side) hindpaw of CCI-rats with a rapid onset of action at 15 min post-dosing. The duration of action of the lipidic derivatives **3** and **4** was 2 h (Fig. 4A, 4B). Compounds **3** and **4** at 0.8 and 0.5 µmol/kg, respectively, were equi-effective with morphine at 2 µmol/kg (Fig. 4C). These results showed the higher potency of compounds **3** and **4** (compared with morphine). Mean (± SEM) ED_50_ values for compounds **3** and **4** were 1.22 (±0.93) µmol/kg and 0.99 (±0.89) µmol/kg, respectively, which was not significantly different (*p*>0.05) indicating both analogs were equi-effective for the relief of mechanical allodynia. ED_50_ values were calculated from the dose-response curves shown in Fig. 5. Given that Dmt is significantly more expensive and time-consuming to produce than tyrosine, the Tyr analog **3** was considered to be a more viable candidate for drug development. Thus, further experiments on the lipidated analogs were performed using compound **3**. Compounds **1** and **2** did not produce significant antinociception at the highest dose tested following i.v. administration (Fig. 6). Antinociception in the contralateral (uninjured) hindpaws of the CCI-rats was not produced after administration of endomorphin-1 derivatives **3** and **4** at the doses tested, in contrast to morphine, which produced significant antinociceptive activity in the contralateral hindpaw (*p*<0.001, Fig. 7).

### Naloxone hydrochloride pretreatment

**Figure 8:**
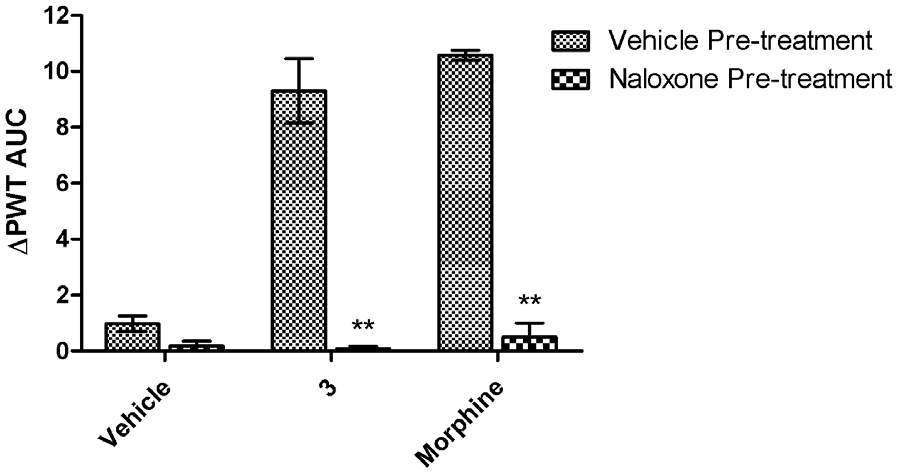
Pre-treatment with the opioid receptor antagonist. The anti-allodynic effects of single i.v. bolus doses of compound **3** were abolished by naloxone (10 mg/kg s.c.) in the ipsilateral hindpaws of opioid-naive CCI-rats (1.6 µmol/kg, *n* = 6) in a manner similar to morphine (2 µmol/kg) Each value is the mean (± SEM). **p<0.01, *vs.* vehicle pre-treated rats (*n* = 6).

The anti-allodynic effects of compound **3** at 1.6 µmol/kg and morphine at 2 µmol/kg were abolished after pre-treatment of CCI-rats with single bolus subcutaneous (s.c.) doses of naloxone hydrochloride, a non-selective opioid receptor antagonist, at 10 mg/kg (Fig. 8, *p*<0.001).

### Constipation inducing properties

**Figure 9:**
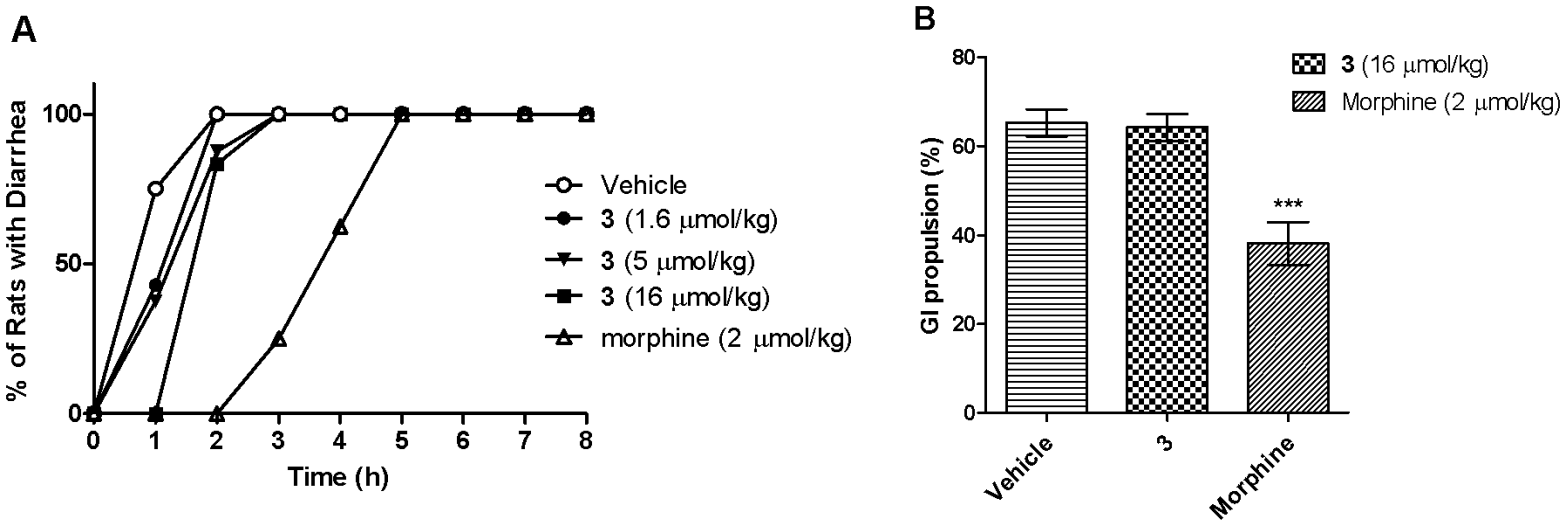
Constipation-inducing activity of compound 3 relative to morphine following single i.v. bolus dose administration. A) Lack of inhibitory effect of **3** in doses up to 16 µmol/kg on castor-oil induced diarrhea in rats in contrast to the significant inhibitory effects of morphine at 2 µmol kg/kg. B) Lack of effect of analog **3** (16 µmol/kg) and vehicle in contrast to the inhibitory effect of morphine (2 µmol/kg) on the gastrointestinal transit of a charcoal meal in rats. Data are the mean (± SEM). ***p<0.001, *vs.* vehicle (*n* = 8).

The effects of the opioid receptor agonist **3** on the gastro-intestinal (GI) transit and stool hydration were investigated using the charcoal test and the castor oil-induced diarrhea assay, respectively. These tests assessed the constipation-inducing propensity of compound **3** and morphine following i.v. administration. No significant inhibition of castor oil-induced diarrhea was produced by compound **3** in doses up to 16 µmol/kg (*p*>0.05). By contrast, i.v. doses of morphine at 2 µmol/kg inhibited castor oil-induced diarrhea significantly, *p*<0.001 (Fig. 9A). Similarly, there was an insignificant effect on the gastrointestinal transit of a charcoal meal following i.v. administration of the highest dose of compound **3** (16 µmol/kg). The mean (± SEM) GI propulsion (%) was 64.3 (±3.0) for rats treated with analog **3**, which was not significantly different (*p*<0.05) from that observed for vehicle-treated animals (65.3±2.4). This was in contrast to the markedly decreased gastrointestinal peristalsis produced by morphine at an i.v. dose of 2 µmol/kg (29.5±4.6%, *p*<0.001, Fig. 9B).

### Development of tolerance

**Figure 10:**
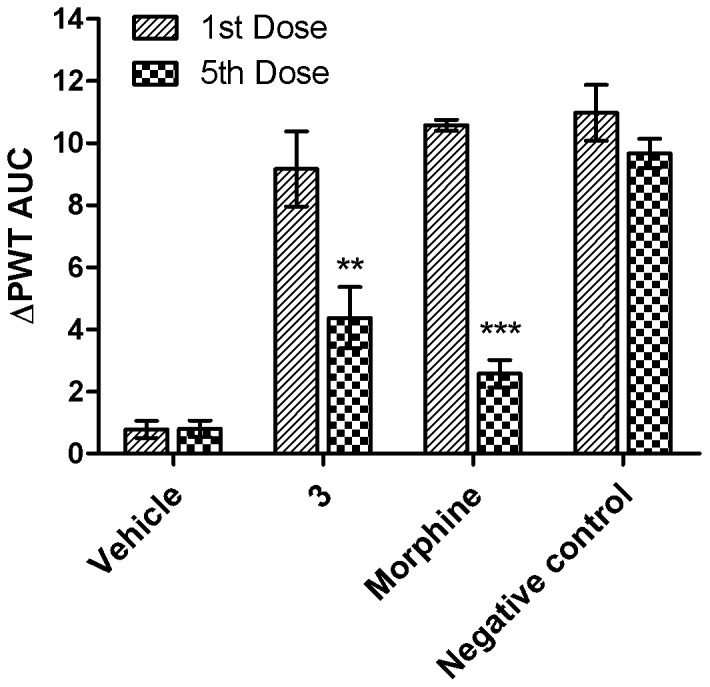
Development of tolerance to the analgesic effect of compound 3 and morphine. Five consecutive i.v. bolus doses of compound **3** (1.6 µmol/kg) or morphine (2 µmol/kg) were administered twice-daily to CCI-rats. The mean (± SEM) ΔPWT AUC values were significantly lower for the 5^th^ dose c.f. the 1^st^ dose, demonstrating that antinociceptive tolerance had developed; however, the extent of tolerance developed to the analgesic effects of compound **3** was lower than that of morphine. There was no evidence of antinociceptive tolerance in the negative control group rats for treated with compound **3** for the 1^st^ dose followed by vehicle for doses 2–4 and then compound **3** for the 5^th^ dose. Data are the mean (± SEM). **p<0.01, ***p<0.001, *vs.* 1^st^ dose of the compounds (*n* = 8).

Development of tolerance to the pain-relieving activity of compound **3** was assessed. I.v. bolus injections were made twice-daily at 1.6 µmol/kg for 5 consecutive doses in naive CCI-rats. Tolerance to the pain-relieving effects of morphine at 2 µmol/kg was examined under the same protocol for comparison. Tolerance developed to the pain-relieving effects of compound **3** (*p*<0.01) as well as morphine in CCI-rats. However, the extent of tolerance development for compound **3** was lower than for morphine. After the 5^th^ dose, the potency of compound **3** had decreased to 48% of that determined after the 1^st^ dose while the potency of morphine had reduced to 24% of that of the 1^st^ dose (Fig. 10).

## Discussion

Similar to the parent compounds **1** and **2**, LAA-modified derivatives **3** and **4** showed moderate to high MOP receptor affinity, which was consistent with their *in vitro* functional activity as agonists at these receptors. In *in vivo* experiments, both compounds **3** and **4** produced marked dose-dependent pain relief following i.v. administration to CCI-rats, which was blocked by pre-treatment with naloxone, consistent with expectations for an opioid agonist. These findings strongly indicate the essential role of opioid receptors in mediating the anti-neuropathic activity of the peptide analogs. Interestingly, both LAA-conjugated endomorphin-1 derivatives **3** and **4** were more potent than morphine for the relief of mechanical allodynia in CCI-rats when administered by the i.v. route.

Endomorphins like other small peptides are degraded rapidly by membrane-bound exo- and endopeptidases in the blood [22] and also enzymes in the BBB, which inhibit their penetration into the brain [23]. Furthermore, due to their hydrophilicity, endomorphins are unable to cross the BBB by transmembrane diffusion [24]. It has been previously shown that permeation of peptides across the BBB can be improved by increasing lipid solubility [25], [26]. This in turn enables peptides to cross the BBB via passive diffusion, a nonsaturable mechanism largely dependent on lipid solubility [27]. Taken together, potent *in vivo* analgesia produced by compounds **3** and **4**, support *in vitro* results demonstrating improved stability of the peptide analogs and enhanced membrane permeability due to an increase in lipophilicity. According to the biopharmaceutics classification system (BCS), Caco-2 cell permeability coefficients (*P*_app_ values) and fractional absorption values (Fa) in humans are highly correlated. In this comparison, it was shown that for compounds with high Fa (%), the corresponding *P*_app_ values are in the range, 10^−5^–10^−6^ cm/s [28], [29]. Based on this classification, LAA-modified derivatives **3** and **4** are within the range of permeable compounds across barriers like the BBB. The Caco-2 cell model has been shown to be a reliable model when studying CNS targeted compounds, for either passive or active transport across the BBB [30], [31].

It was shown that introduction of Dmt into the structure of endomorphin-1 derivatives not only enhanced MOP receptor binding affinity, as reported previously [11], but also enhanced the agonist functional activity of the peptides *in vitro*. However, replacement of the Tyr residue with Dmt had an insignificant impact on the *in vivo* pain relieving potency (*p*>0.05), leaving analog **4** of less value for further pharmacological investigation. The higher *P*_app_ value obtained for compound **3** compared with analog **4** may be responsible for a better permeability of this compound across the BBB. This higher permeability could offset the lower binding affinity of compound **3** to produce similar analgesia *in vivo.* Moreover, although *in vitro* biological studies are useful tools in the development of therapeutically active peptides, combined use of both *in vitro* and *in vivo* methods is essential to assess the activity of these modified peptides. Between-analog differences in pharmacokinetic (metabolism, volume of distribution, elimination half-life, protein binding and systemic clearance) and pharmacodynamic (tissue selectivity, receptor binding) and physicochemical (peptide aggregation) factors [32], all have the potential to influence net activity observed *in vivo*.

Morphine produces antinociception *via* activation of the endogenous descending inhibitory system [33] with anti-allodynia/antinociception produced in the ipsilateral hindpaw and antinociception in the contralateral hindpaw of CCI-rats. Furthermore, our results showed that the effect of morphine on the ipsilateral hindpaw of the CCI-rats is not statistically different from the effect on the contralateral paw, which was consistent with previous reports in the literature [34], [35]. In contrast the anti-allodynic effects of compound **3** and **4** were selective to the ipsilateral hindpaw with insignificant effects produced in the contralateral hindpaw. These observations were in agreement with a previous report, where continuous intrathecal administration of peptide **1** produced selective anti-hyperalgesic responses in the inflamed hindpaw of a carrageenan-induced inflammatory pain model of rat [22].

A common side-effect produced by MOP-receptor agonists is constipation, which occurs because of a reduction in stool hydration and delayed GI transit [7]. In the present study, i.v. bolus doses of compound **3** produced insignificant inhibition of castor oil-induced diarrhea and no significant delay on GI transit in the charcoal test at 16 µmol/kg. On the contrary, i.v. bolus doses of morphine at 2 µmol/kg significantly inhibited castor oil-induced diarrhea and delayed GI transit in the charcoal test.

Our findings showed that less tolerance developed to the analgesic effect of compound **3** compared to morphine. There are several reports on the development of tolerance to endomorphins after systemic administration [36]–[38]. The extent of tolerance developed by a MOP receptor agonist is dramatically affected by pharmacokinetic factors [25], duration of opioid receptor occupancy (which in turn is relative to the duration of action), and the dosing interval of the administered compound [39].

## Conclusion

We have successfully designed and developed two systemically active lipid-modified derivatives of endomorphin-1 with promising potential for the treatment of neuropathic pain. Lipid derivatives **3** and **4** were more potent than morphine. Analog **3** is a systemically active derivative of endomorphin-1 exhibiting potent analgesic activity without constipation, a major side effect associated with opioid use. This is a highly novel finding in the opioid analgesic field. Furthermore, compound **3** produced less anti-allodynic tolerance than morphine. This divergence in pharmacological effects of morphine and the lipidic endomorphin-1 derivatives is a significant finding and will be further investigated. In conclusion, lipidic derivatives of endomorphin-1 can be developed as novel opioid analgesic agents for human use to treat neuropathic pain.

## Materials and Methods

### Ethics Statement

This research was approved by and conducted in compliance with the guidelines of The University of Queensland Animal Ethics Committee (AEC#CIPDD/366/09/NHMRC).

### Chemistry

#### General

Dimethylformamide (DMF), trifluoroacetic acid (TFA) and piperidine of peptide synthesis grade were purchased from Merck Biosciences (Kilsyth, VIC, Australia), and acetonitrile suitable for HPLC was purchased from RCI Labscan Ltd. (Bangkok, Thailand). Fmoc-protected amino acids and Rink amide MBHA resin (100–200 mesh) with a loading of 0.4–0.8 mmol/g were obtained from Novabiochem (Melbourne, VIC, Australia) or Mimotopes (Clayton, VIC, Australia), apart from Fmoc-dimethyltyrosine (Fmoc-Dmt) which was sourced from Chiramer, Inc. (USA). Cell culture reagents were purchased either from Sigma Aldrich or Gibco (VIC, Australia). Other chemicals were purchased from Sigma Aldrich (VIC, Australia). Boc-C10-LAA was synthesized according to a published procedure [39]. A Varian Cary 50 Bio UV/Vis Spectrophotometer (λ = 570 nm) was used for absorbance measurements. Preparative HPLC was carried out on a Waters system, equipped with a 600 controller and pump and 490E UV/Vis detector operating at a wavelength of 230 nm. An Agilent 1100 system with binary pump, autosampler and UV/Vis detector set to 214 nm was used for analytical HPLC. Electrospray ionization mass spectrometry (ESI-MS) was performed on a PE Sciex API3000 triple quadrupole mass spectrometer, operating with a constant flow of a 1∶1 mixture of solvent A (0.1% formic acid in water) and B (0.1% formic acid in acetonitrile/water 9∶1) at a rate of 0.05 mL/min.

#### Solid-phase peptide synthesis

Peptides were assembled on Rink amide MBHA resin following the in situ neutralization protocol for Fmoc chemistry [40]. The following protected amino acids (4.2 eq., except Fmoc-Dmt 1.3 eq.) were activated with HBTU (4 eq.)/DIPEA (5 eq.) prior to coupling: Fmoc-Phe, Fmoc-Trp(Boc), Fmoc-Pro, Fmoc-Tyr(tBu), Fmoc-Dmt and Boc-C10-LAA. Coupling was monitored using the chloranil test [41] for proline and the ninhydrin test [42] for all other amino acids, and if the efficiency was below 99.6% recoupling was performed.

Upon completion of the peptide sequence, the resin was washed with DMF, dichloromethane and methanol, and dried under vacuum over night. The peptide was cleaved from the resin using a mixture of TFA (95%), triisopropyl silane (2.5%) and water (2.5%). After 2 hours, the solvent was removed and the peptide precipitated using cold diethyl ether. The resulting solid was lyophilized from acetonitrile, water and TFA (50∶50∶0.1).

#### Purification

Purification of the crude peptides was carried out on a Vydac C18 column (22×250 mm) at a flow rate of 10 mL/min with a 70 minute gradient of 40% to 80% solvent B (0.1% TFA in acetonitrile/water 9∶1; solvent A: 0.1%TFA in water). The collected fractions were analyzed by mass spectrometry and analytical HPLC on a Vydac C18 column (4.6×250 mm, 5 µm) at a flow rate of 1 mL/min and a gradient of 0% B to 100% B over 30 minutes. Pure fractions were combined and lyophilized. Purity of peptides was confirmed by analysis on a different HPLC column (C4, 4.6×250 mm, 5 µm) and high resolution mass spectrometry.

Compound **1** Yield: 108 mg, 84%. Exact mass calculated: 611.2976, found: 611.2987 [M+H^+^].

Compound **2** Yield: 86 mg, 57%. Exact mass calculated: 639.3311, found: 639.3289 [M+H^+^].

Compound **3** Yield: 466 mg, 43%. Exact mass calculated: 780.4443, found: 780.4471 [M+H^+^].

Compound **4** Yield: 450 mg, 40%. Exact mass calculated: 808.4734, found: 808.4756 [M+H^+^].

### In vitro experiments

#### Cell culture

Cell lines were obtained from the American Type Culture Collection (Rockville, USA). Human colorectal adenocarcinoma (Caco-2) cells were maintained in T-75 flasks with culture medium consisting of Dulbecco's modified Eagle's medium (DMEM) supplemented with 10% fetal bovine serum (FBS), 1% L-glutamine, and 1% non-essential amino acids. Flasks were kept in an incubator at 95% humidity and 37°C, in an atmosphere of 5% CO_2_. The culture medium was changed every second day. Upon reaching 80% confluence, cells were subcloned using 0.25% trypsin–EDTA (ethylenediaminetetraacetic acid). Experiments were performed using cells with passage numbers 55–75. Human neuroblastoma (SH-SY5Y) cells were maintained in DMEM:Hams F12 medium supplemented with 10% FBS, 1% glutamine, 1% non-essential amino acids and 1000 U/mL Streptomycin/Penicillin. Cells were subcloned in the same manner as Caco-2 cells, and passage numbers 10–20 were used in the assays.

#### Caco-2 cell homogenate stability assay

A suspension of 5×10^4^ Caco-2 cells in 100 µL culture medium was added to the wells of a 96-well cell culture plate. Culture medium supplemented with 1% penicillin/streptomycin (100 U/mL) was changed every second day.

After 21–28 days, the cells were washed with 0.2% EDTA (2×100 µL) and Hank's Balanced Salt Solution containing 25 mM 4-(2-hydroxyethyl)-1-piperazineethanesulfonic acid (HBSS-Hepes, pH 7.4; 3×100 µL). HBSS–Hepes buffer was added to each well (100 µL), and the cells were disrupted by two one-second pulses with a Sonics Vibracell ultrasonic processor set at 60% amplitude at 130 W. The cell debris was removed by centrifugation (2000 rpm, 5 minutes) and the protein content of three wells was determined using the Bio-Rad protein assay kit, by comparison with a standard curve generated from known concentrations of bovine serum albumin. The protein content of the cell homogenate was adjusted to 0.6–0.9 mg/mL in a clean 96-well plate.

The peptides were dissolved in HBSS–Hepes buffer containing 1% DMSO (dimethyl sulfoxide), and each peptide solution (200 µM, 100 µL) was added to three wells and the plate was incubated at 37°C in a shaker at 400 rpm. At pre-selected time points (5, 10, 15, 20, 30, 40, 50, 60, and 120 min), samples (10 µL) were collected and enzymatic digestion was stopped by addition of 5 µL TFA. These samples were then diluted with water (75.5 µl) and DMSO (9.5 µL) and analyzed by LC-MS to determine the concentration of peptide remaining in the solution.

LC-MS was carried out on a Luna Phenomenex C18 column (2.0×50 mm, 5 µm) with a gradient of 100% A (0.1% formic acid in water) to 100% B (0.1% formic acid in acetonitrile/water 9∶1) over 5.5 minutes at a flow rate of 0.3 mL/min. The mass spectrometer was set to selective ion monitoring in the positive ion electrospray mode. A standard curve was generated from standards containing known peptide concentrations, and used to determine the peptide concentration in the samples.

#### Caco-2 cell permeability assay

A suspension of 10^5^ Caco-2 cells (100 µL) in culture medium supplemented with 1% penicillin/streptomycin (100 U/mL) was pipetted into polycarbonate cell culture inserts (pore size 0.4 µm, 6.5 mm diameter, Transwell®) in a 24-well plate, and the same medium was also added to the basolateral chamber (0.6 mL). Medium in both chambers was changed every second day.

After 21–28 days, the integrity of the tight junctions and the monolayers was assessed by measuring the transepithelial electrical resistance (TEER) using the Millicell-ERS epithelial volt-ohmmeter system (Millipore Corporation). Wells that gave TEER values of 1500–4280 (Ωcm^2^) were used for the experiment. Minor TEER value differences of ±500 (Ωcm^2^) were detected after the assay, indicating that none of the peptides were toxic to the cells.

After the initial TEER test, the Caco-2 cell monolayers were washed with HBSS-Hepes buffer (3×) and then incubated with the same buffer at 37°C. After 30 minutes the buffer was removed from the apical chamber and 100 µL aliquots of peptides, dissolved to a concentration of 200 µM in HBSS-Hepes containing 1% DMSO, were added to at least three wells. As a further test of the Caco-2 cell monolayer integrity, radiolabelled [^14^C]-D-mannitol (1.80 µCi) in HBSS-Hepes buffer was added to three wells. The plates were incubated in a shaker set to 400 rpm at 37°C, and samples (0.4 mL) were collected from the basolateral chamber at 30, 90, 120 and 150 minutes and replaced with the same volume of buffer. At the end of the experiment, 50 µL were collected from the apical chamber of each well. LC-MS quantification of the peptides was performed as described in Caco-2 cell homogenate stability assay. The radioactivity of the [^14^C]-D-mannitol samples was quantified by liquid scintillation counting (Liquid Scintillation Systems, BECKMAN LS3801, USA), and the apparent permeability (*P*_app_) for each compound was calculated using the following equation:
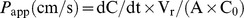
dC/dt steady-state rate of change in the chemical concentration (M/s) or radiochemical concentration (dpm mL/s) in the receiver chamber

V_r_ volume of the receiver chamber (mL)

A surface area of the cell monolayers

C_0_ initial concentration in the donor chamber (M or dpm/mL)

#### Plasma stability assay

Fresh human plasma was obtained from healthy and consenting volunteers (ethics approval number: 2006000950). The peptides were dissolved in PBS containing 5% DMSO (1 mg/mL), and 300 µL aliquots were mixed with 300 µL of pre-warmed plasma and incubated at 37°C. At selected time points (5, 10, 20, 30, 40, 60, 90, and 120 min), samples (50 µL) were collected and plasma proteins precipitated by addition of acetonitrile (75 µL). After centrifugation (10,000 rpm, 10 minutes), the peptide content of the supernatant was analyzed by HPLC with a Vydac C18 column (4.6×250 mm, 5 µm) at a flow rate of 1 mL/min and a gradient of 100% A (0.1% TFA in water) to 100% B (0.1% TFA in acetonitrile/water 9∶1) over 30 minutes.

#### SH-SY5Y receptor binding assay

A suspension of SH-SY5Ycells (10^5^ cells per well) was seeded into 24-well plates and incubated overnight. The cells were washed with PBS and pre-incubated with 300 µL binding buffer (50 mM Tris buffer, pH 7.4, 2% BSA). A mixture of [^3^H]-DAMGO (100 µL, 15 nM), DPDPE (100 µL, 1 µM) and 100 µL aliquots of each peptide in a range of concentrations (0.1 nM to 100 µM) was added to each well and the plate was incubated at 37°C. An excess of [^3^H]-DAMGO (1 µM) was used to determine non-specific binding and buffer (100 µL) was measured as the blank. After 60 minutes, the cells were washed with PBS and 1 M NaOH (0.5 mL) was used to lyse the cells. The radioactivity of the cell suspension was measured by scintillation counting (Liquid Scintillation Systems, BECKMAN LS3801, USA). Data analysis was performed using the one-site competition binding model (GraphPad Prism™ (v5.0) software).

#### Inhibition of cAMP accumulation

SH-SY5Y cells, which express MOP and DOP receptors, were seeded (10^5^ cells/mL, 100 µL) in a 96-well plate and incubated overnight to reach 80% confluence. Culture medium supplemented with 30 µM forskolin was used make peptide solution (100 nM) and aliquots (100 µL) of each peptide were added to three wells. After incubation at 37°C for 30 minutes, the culture medium was removed and lysis buffer from a cAMP Biotrak EIA kit (Amersham, Buckinghamshire, UK) was added, followed by an anti-cAMP antibody. After incubation at 4°C for 2 hours, a cAMP peroxidase conjugate was added and the plate incubated for another hour at 4°C. Then, the cell lysate was incubated with a 3,3′5,5′-tetramethylbenzidine (TMB) substrate provided by the Biotrak EIA kit and finally 1 µM sulfuric acid was added. Absorbance of each well was determined using a plate reader at 450 nm and compared with a cAMP standard curve (concentration 0–3.2 pmol). Data was represented as a percentage of the maximum cAMP response to forskolin, shown as a mean (±SEM) of three separate experiments each performed in triplicate (GraphPad Prism™ (v5.0) software).

### In vivo experiments

#### Animals

Adult male Specific Pathogen Free (SPF) Sprague-Dawley (SD) rats weighing between 160–200 g were purchased from The University of Queensland Biological Resources breeding facility. They were housed in groups of 3 with free access to food and water in a light- and temperature-controlled room (light on 06:00–18:00 h; 22.2±0.2°C) and 51.6–65.2% humidity. Prior to initiation of experimental procedures, rats were acclimatized for at least a 3-day period.

#### Experimental model of nociception

Chronic constriction injury (CCI) of the sciatic nerve in the rat was used as a model of neuropathic pain and was induced according to the method of Bennett and Xie [21]. Briefly, rats were anesthetized with 3% isoflurane delivered in oxygen. A 7 mm segment of the left common sciatic nerve was exposed at mid-thigh level, proximal to the sciatic trifurcation. Four silk sutures were tied loosely around the nerve at intervals of 1 mm. The surgical incision was closed by suturing the muscles and skin in layers. To prevent infection, topical antibiotic powder was applied to the wound. The day of CCI surgery was regarded as day 0. Rats were inspected daily considering posture of the affected hindpaw, exploring behavior, body weight, water intake, and signs of autotomy until experimentation day, which was 14 days post-surgery.

#### Nociceptive test: Assessment of Von Frey paw withdrawal thresholds

Paw withdrawal thresholds (PWT's), expressed in grams, were measured using a set of calibrated Von Frey filaments (Touch Test- North Coast, 800-821-9319). Animals were acclimatized for at least 15 min in wire mesh metabolic cages (20×20×17 cm). Von Frey filaments which delivered forces in the range 2–20 g at 2 g intervals were used according to the up-down method [20]. Filaments were applied to the plantar surface of the footpad of the hindpaw so that the filament bent slightly for 3 seconds. Every trial was commenced with the 6 g Von Frey filament and the force was increased progressively at 2 g intervals until the animal withdrew its paw, or until a maximum force of 20 g was reached. Rats not responding to any of the filaments were arbitrarily assigned a PWT value of 20 g. If the rat responded, the next filament in a descending sequence was tested. Fourteen days after surgery, full mechanical allodynia, defined as PWTs ≤6 g, was developed in the ipsilateral (operated side) hindpaw.

#### Test and control item administration

Test items were formulated in 10% DMSO (Sigma-Aldrich) and 20% Cremophor (Sigma-Aldrich) in water and were administered as i.v. bolus doses via tail-vein injection. Morphine sulphate (Hospira, Melbourne Australia) was used as the positive control and vehicle as the negative control in this study. In accordance with animal ethics guidelines for reduction of the number of animals used in experiments, the PWT values for the negative and positive controls shown here have also been included in another manuscript (P. Varamini et al. 2012, submitted). Test and control items were administered to groups of CCI-rats according to a ‘washout’ protocol such that each rat received 3–4 single i.v. bolus doses in a rising dose sequence, of one test item or vehicle with each dose separated by a 2–3 day washout period. On each dosing occasion, Von Frey PWTs were determined in the ipsilateral and contralateral (non-operated side) hindpaws immediately prior to test item administration (baseline PWT values) and at the following times post-dosing: 0.25, 0.5, 0.75, 1, 1.25, 1.5, 2, and 3 h.

#### Naloxone-sensitivity of antinociception/anti-allodyfnia

Groups of CCI-rats (*n* = 6) were pre-treated with single subcutaneous bolus doses of either vehicle or naloxone hydrochloride (10 mg/kg) at 5 min prior to i.v. administration of single bolus doses of compound **3** (1.6 µmol/kg), morphine (2 µmol/kg) or vehicle. PWTs were determined just prior to vehicle or naloxone administration and at the following times after test item administration: 0.25, 0.5, 0.75, 1, 1.25, 1.5, 2, and 3 h.

#### Assessment of constipation inducing properties

Assessment of constipation inducing properties:

1. Castor oil-induced diarrhea test in rats
  The ability of test items to inhibit castor-oil induced diarrhea as an index of constipation was assessed. Prior to the start of experimentation, drug-naive rats weighing 200–250 g were fasted for 16 h with free access to water. Individually caged rats were acclimatized for 1 h without access to food or water prior to drug or vehicle administration. Compound **3** (1.6, 5 or 16 µmol/kg), morphine (2 µmol/kg) or vehicle were administered as single i.v. bolus doses via the tail vein. Rats were gavaged with 1 mL of castor oil five minutes after test item or vehicle administration. Presence or absence of diarrhea was recorded every hour according to a scoring system based on the weight of feces boli and the feces consistency. Weight was scored from 0 (no feces) to 3 (>3 g of feces) and consistency was scored from 0 (normal-shape feces) to 3 (shapeless feces with large amounts of liquid). When both weight of feces and the feces consistency were scored at ≥2, diarrhea was considered to be present.
2. Charcoal test
  The charcoal test was assessed in groups of drug-naive rats to measure gastrointestinal (GI) transit. Prior to experimentation, rats (200–250 g) were fasted for 16 h with free access to water. Single i.v. bolus doses of compound **3**, morphine or vehicle was administered to naive rats (*n* = 8 per group). Subsequently, 1 mL of 10% aqueous activated charcoal and 5% gum Arabic was administered by oral gavage to rats. Rats were euthanized with i.p. sodium pentobarbital (65 mg per rat) one hour later followed by cervical dislocation. The intestine was carefully removed and separated from omentum whilst avoiding stretching. The total length of the small intestine from the pylorus to the ileocaecal junction as well as the most distal point of migration of the charcoal meal was measured. The percentage of the distance which the charcoal meal travelled relative to the total length of the small intestine (GI propulsion) was calculated.

#### Assessment of antinociceptive tolerance

At 14 days after CCI surgery, rats (350–400 g) were randomly assigned to one of four treatment groups and administered twice-daily i.v. bolus doses of one test item or vehicle for 5 consecutive doses. Group 1 CCI-rats received compound **3** at 1.6 µmol/kg for all 5 doses whereas group 2 CCI-rats (negative control) received compound **3** at 1.6 µmol/kg followed by vehicle for doses 2–4 with the 5^th^ dose comprising compound **3** at 1.6 µmol/kg. Groups 3 and 4 CCI-rats received 5 consecutive doses of vehicle or morphine (2 µmol/kg), respectively. Paw withdrawal thresholds were measured immediately pre-dose and at the following post-dosing times, viz 0.25, 0.5, 0.75, 1, 1.25, 1.5, 2, and 3 h for doses 1 and 5.

#### Data analysis

To obtain ΔPWT values, the PWT versus time data were normalized by subtracting pre-dosing baseline PWT values for each individual rat as follows:
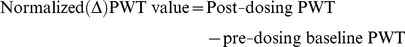
ΔPWT values were used to obtain the extent and duration of antinociception/anti-allodynia. The extent and duration of antinociception/anti-allodynia (area under the ΔPWT versus time curves; ΔPWT AUC values) was estimated using trapezoidal integration for individual CCI-rats. Mean (± SEM) ΔPWT AUC versus log dose curves were constructed and the AUC values were normalized relative to the maximum possible ΔPWT AUC value to give % Maximum Possible Effect (% MPE) values. Non-linear regression as implemented in the GraphPad Prism™ (v5.0) software program (GraphPad Software) was used to estimate the ∼ED_50_ values.

#### Statistical analysis

All data are expressed as mean (± SEM). One-way ANOVA was used to compare normalized ΔPWT AUC values of each LAA-conjugate between the ipsilateral and contralateral hindpaws in addition to GI propulsion and castor-oil induced diarrhea inhibition produced by compound **3** and morphine, compared with that of vehicle. All comparisons between two treatment groups including comparisons between ΔPWT AUC values of naloxone and vehicle pretreated CCI-rats and the ED_50_ values for compounds **3** and **4,** were performed using the non-parametric Mann-Whitney test as implemented in the GraphPad Prism™ (v5.0) software package. The statistical significance criterion was *p*<0.05.

## Supporting Information

Scheme S1 Synthesis of endomorphin-1 (1) and its derivatives (2–4). (i) 20% piperidine/DMF, 0.5 h (ii) AA (1. Fmoc-Phe-OH 2. Fmoc-Trp(Boc)-OH 3. Fmoc-Pro-OH 4. Fmoc-Tyr(tBu)-OH or Fmoc-Dmt-OH), HBTU, DIPEA, DMF, rt, 1.5 h (iii) HBTU, DIPEA, DMF, rt, 1.5 h (iv) TFA, triisopropylsilane, H_2_O, rt, 2 h.
(TIF)
